# Ipsative Assessment and Peer Feedback in an Orthopaedic Junior Doctor Teaching Programme: A Project Plan and Narrative Review of the Literature

**DOI:** 10.7759/cureus.31961

**Published:** 2022-11-28

**Authors:** Fitzgerald Anazor

**Affiliations:** 1 Trauma and Orthopaedics, William Harvey Hospital, Ashford, GBR

**Keywords:** narrative review, peer feedback, ipsative assessment, clinical teaching, orthopaedics, project plan

## Abstract

The main aim of this study is to describe a plan for a project to introduce the use of formative ipsative assessment and peer feedback within an orthopaedic junior doctor teaching programme. These changes will ensure that students demonstrate objective progress and understand that they are making progress in their learning journey. It will also improve learner collaboration through the creation of communities of practice. Key stakeholders involved include the junior doctors, tutors, consultants, medical education department, research/audit department, and orthopaedic company representatives. Outcomes will be measured using a four-point Likert scale on Google forms digital portfolios for the domains of clinical knowledge, technical skills, communication, teamwork, and basic research skills. Progress will be audited at six-month intervals. Full project implementation will be within 6-12 months. A narrative review of relevant literature and theories of learning in relation to ipsative assessment and peer feedback within a clinical teaching context was also performed.

The future of medical education will still contain large components of ipsative assessment. In addition, a problem-based collaborative learning approach is now utilized in many medical schools and peer feedback will become more frequently utilized as a part of this in future. The author looks forward to implementing this project successfully and anticipates that the knowledge/skills gained from this will be useful for any future career projects both within and outside medical education.

## Introduction and background

The index teaching programme involves near-peer junior doctor teaching sessions in trauma and orthopaedics with a class size of 7-15 students per session. This is a blended programme with mainly face-to-face sessions with a virtual/e-learning component based on an outcome-based learning approach. Sessions are delivered once weekly and last 45-60 minutes. The overall learning objectives of the programme are to improve the clinical knowledge and general professional capabilities (domains like communication, teamwork, basic research skills, technical skills, and professional integrity) of learners to a level expected of a day one speciality trainee 3 (ST3) doctor or registrar in trauma and orthopaedics. 

There is a wide learner and tutor diversity in terms of race, gender, and country of primary medical qualification with a mix of United Kingdom-trained doctors and international medical graduates. There is also diversity in learner experience/skills ranging from foundation-year doctors who are one to two years post graduation to more senior registrar-level doctors (above four years post graduation). Acknowledging and catering for this learner diversity is important in order to deliver a sound, encultured learning programme as baseline learner beliefs and knowledge can influence the perception of any new information and taught skills, the “theory of constructivism” [1]. With the current notion of internationalization in education, catering to the diverse learning needs in this learning cohort is very important in order to foster an inclusive and equitable National Health Service (NHS). Junior doctors usually rotate through the department every 6-12 months on average. This means there could be a sustainability issue for any quality improvement initiative one engages in. Thus, measures to institutionalize any programme initiative are invaluable towards addressing this potential drawback. Teaching and learning on the programme are voluntary with no funding available from tuition fees or research endowments. Thus, any changes implemented will have to be relatively simple, cost-effective, and delivered within the time constraints of a clinical teaching setting. 

A lack of an objective and collaborative assessment of students’ progress in their learning journey during the teaching program was identified as a problem because assessment of learning progress is a key pillar of a successful teaching and learning program. Progress in learning can be assessed either by the tutor, the learners, or peers. Assessment of learning can be done in an ipsative manner or through standard means either using formative or summative techniques. Learners being aware of progress made will serve to enrich the learning process and potentially motivate them towards future learning goals. Furthermore, an objective learning assessment relies on a constructive alignment of learning outcomes with learning assessment [2]. A good program is also based on an excellent understanding of learners’ baseline knowledge and beliefs, which should influence learning design. This constructivism theory by Rillo et al., states that “students participate in the construction of knowledge during the educational process with their life experience and a preconceived cognitive structure based on it, which is associated with memory” [3]. 

The main aim of this planned project is to institute the use of ipsative assessment and peer feedback within the aforementioned teaching programme. These changes will ensure that students demonstrate objective progress and understand that they are making progress in their learning journey. This will also provide an understanding of the levels of achievement of the overall learning objectives of the teaching programme to both the students and the tutors.

This project was part of a final assessment of the master’s level postgraduate certificate in higher education at the University of London, London, United Kingdom.

## Review

Project plan

The new measures that will be introduced are as follows: (A) Introduction of an ipsative assessment method for the core clinical knowledge and skills domains (teamwork, communication, research, technical skills and knowledge) and (B) Introduction of peer-review workshops for peer feedback of learning progress. 

The new programme modifications will be introduced for the next cohort of junior doctors who will commence their rotation in April 2023. It will involve a pilot model for the first four weeks followed by an assessment of the strengths and limitations via feedback from learners and tutors and further programme modification as required. Finally, there will be further assessment of the quality improvement objectives at six months and 12 months following the implementation of the project plan. 

Performance indicators and assessment of key milestones will involve the use of a four-point Likert scale to assess the aforementioned professional capabilities and skills. However, feedback will still be collated for each session as per current practice. Key project quality improvement targets will be at least a 75% engagement of the learners on the peer review workshops and demonstrable improvement by 100% of learners after six months for the key general professional skill domains mentioned previously. 

The ipsative assessment will be implemented by assigning a four-point rating to the level of knowledge/skills exhibited by the learner similar to the rating on the intercollegiate surgical curriculum programme portfolio [4], but different in the fact that the maximum target is performance to the level of a speciality trainee 3 (ST3) registrar rather than that of a consultant. Level 1 implies an ability to describe basic elements of the topic or assist on a practical element with basic proficiency. Level 2 is for a learner that demonstrates a good understanding of the topic and is able to demonstrate practical or professional skills with no fluency. Significant tutor guidance is required at this level. Level 3 is a demonstration of higher levels of analysis/evaluation of clinical issues or performance of professional/technical skill with some fluency under direct supervision. Level 4 is the demonstration of an excellent critical evaluation of clinical/research issues with proficiency in professional/technical skills to that expected of an ST3 registrar under direct supervision. These will be collated in real time for each student via separate self, peer, and tutor assessments. Each component of these three assessments will be collated on digital portfolios using Google Forms (Google LLC, Mountain View, California, United States) at the end of each learning session and then added to a digital portfolio created for each student either on the Intercollegiate Surgical Curriculum Programme (ISCP) portfolio (for surgical trainees on this platform) or retained on Google Forms (for foundation doctors and non-trainees who do not use the ISCP portfolio). This can be accessed by both the learners and tutors and will be collated continuously to show progress for each learner. On the digital portfolio, progress will also be displayed on a graph to provide a quick pictorial snapshot of the learning journey. There will be assessments for the following key domains: technical skills, research competency, communication skills, clinical knowledge, teaching skills, and professionalism. 

**Figure 1:**
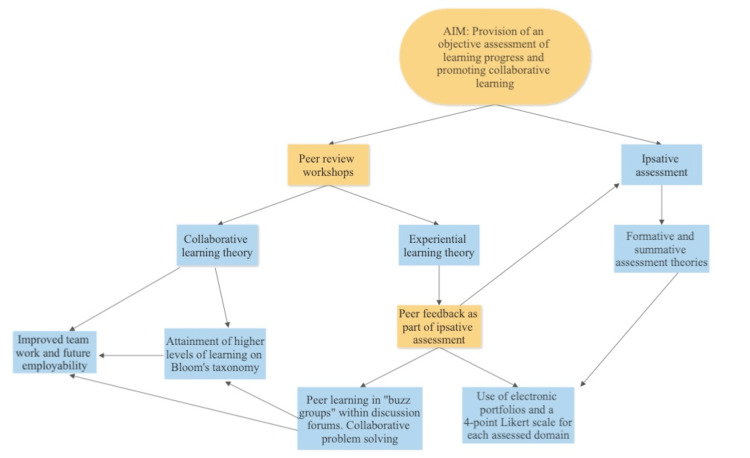
Flow chart summarizing the interconnections between the project aims, underpinning theories, practical components, and learning outcomes

The peer review workshops will mainly be face-to-face sessions where learners will be divided into small "buzz groups" of three to four students per group. Each learner will log into the Google Forms or their ISCP portfolio using a link provided by the tutor for this exercise. For example, if a learner is to discuss a particular clinical topic like “management of ankle fractures”, the colleagues in the group will provide a rating between 1 and 4 as discussed earlier for the relevant domains, which, in this case, will be communications skills, professionalism, teaching skills, and clinical knowledge. This will be repeated for each learner in the mini-group with the tutor providing supervision for all the student groups. To save time and improve efficiency, different sections of the topic in question can be allocated to each student in the mini-group. Apart from using the peer review workshops for assessment of learning, these workshops will be an avenue for students to come together and share ideas on the allocated clinical problem in a problem-based learning approach. Thus, different pedagogical components will come to play in these sessions in order to maximize the learning gains. Finally, the ratings from these peer review assessments will make up a key component of the ipsative assessment as described earlier. Figure 1 summarizes the interconnections between the aims, theoretical basis, and practical components of this project. 

Key Stakeholders Required 

For this plan to be successfully implemented, key stakeholders have been identified, some of whom have been involved in preliminary discussions relating to this project with useful feedback received. These stakeholders include the following: 

Tutors and students on the programme: Drawing on the lessons learnt from the first eight months of the index teaching programme, it has been agreed that peer review workshops and ipsative assessments will be useful to enhance teaching and learning on the programme. Many of the students were excited with the idea of the use of peer review assessments and workshops in a face-to-face or virtual format. Some students felt it would make discussions more robust and provide useful insight from colleagues in a way that is slightly different from the usual tutor feedback. They also felt it provided a feeling of “inclusion and ownership” of the learning experience. All students were familiar with the use of the ISCP portfolio and felt the ipsative assessments could be seamlessly implemented using this platform. 

Departmental clinical lead and consultants: These senior colleagues usually offer direct or indirect supervision for teaching sessions. Support from them will be crucial as most resources for this departmental teaching programme are provided by the department. Consultants will also help liaise with industry implant manufacturers to provide learning tools and orthopaedic models for some of the practical teaching sessions. Finally, they are the ones that usually sign off the ISCP portfolio assessments following student and tutor input. 

Medical education department: Guidance and supervision from the medical education department will be sought before project implementation following preliminary discussions. They have agreed to provide relevant information technology support for the teaching programme. Further discussions are in progress to channel a part of the annual educational budget for junior doctors in order to fund interested tutors to attend a “teach the teachers” course, which will potentially help improve their teaching skills. Furthermore, the two medical education fellows (who will be working in both medical education and orthopaedics) will become tutors on the teaching programme, thus bringing unique skill sets to enrich the learning experience. 

Research and audit departments: Audit and research are important elements of any improvement project. The planned audits of the teaching programme to gauge the achievement of set standards will have to be registered and approval obtained from the audit department. Publications of future research related to this programme will also need collaboration with the research department for study approval, guidance and support with any required resources. 

External stakeholders: Orthopaedics and trauma surgery is a surgical field where industry stakeholders play a major role. Liaison with them to provide simple simulators and kits that will be suitable for teaching junior doctors on the programme will be required. Progression of practical skills by junior doctors is an area where ipsative assessment will play a major role as an assessment tool outside the pressured environment of the operating theatre. Tutors and learners will also be able to provide feedback to the industry in order to improve implant and tool kit design for future models. 

Discussion in relation to relevant literature

By introducing ipsative assessment in this teaching program, learners will be able to assess their progress in a less-pressured and less-competitive manner. Ipsative assessment is based on both formative and summative assessment theories depending on the context [5]. Ipsative assessment will mainly be employed “for learning” based on a formative assessment theoretical basis towards measuring personal learning gain. Ipsative feedback can potentially boost learner self-esteem and motivation [6]. On the other hand, because it is expected to be motivational and developmental, some learners may feel disappointed if further assessments do not lead to improved grades, especially when used in a summative setting. As seen in the study by Hughes et al., this raised expectation could be a demotivational factor for students engaged in the ipsative assessment process if not managed appropriately by the tutor [7]. Another potential drawback of ipsative assessment is that “high achievers” might not see the benefit in this type of assessment, especially if the marks will not count towards a summative assessment. This problem can be tackled by setting a clear structure and objective at the beginning of the learning programme and encouraging these learners to see that there is always room for further improvement. Furthermore, formative ipsative assessment offers the possibility of each learner continuously challenging themselves to become better rather than just “coasting” at a relatively comfortable level. 

The key domains of teamwork, communication, research skills, clinical knowledge, and technical skills all feed into the general professional capabilities of the General Medical Council [8]. These so-called “soft skills” have been identified as key domains for all medical doctors irrespective of their speciality. These skills will also potentially enhance the doctors’ “employability”. This is supported by the study conducted by O’Leary in which nine out of every 10 learners surveyed wanted employability to have a greater emphasis on training and learning [9]. 

Introduction of peer-review workshops for peer feedback is another potential way of improving learning and was found to be very useful following the author’s experience of this as a learner on the Learning and Teaching in Higher education (LTHE) postgraduate certificate of the University of London. Peer feedback conforms to the principles of good feedback according to Nicol and McFarlane-Dick [10], “encouraging peer dialogue, stimulating positive motivational beliefs and promoting critical reflection among peer learners”. 

The first key pedagogic principle underlying peer feedback via peer review workshops is collaborative learning. Collaboration can potentially stimulate deep learning and the teamwork skills gained can improve employability [11]. Collaboration among peers can help engender "communities of practice" for learning [12]. Evidence in the literature points to the advantages of peer feedback in medical education especially if peers are provided with some training or a structured format on how to provide quality feedback [13]. Peer feedback also enhances learner collaboration and teamwork, both in distance learning programmes and in face-to-face sessions. A randomized controlled trial by Simonsmeier et al. showed improved academic self-concept amongst psychology students who were actively engaged in peer feedback for academic writing when compared to a control group [14]. Furthermore, a systematic review by Lerchenfeldt et al. found that 10 out of the 12 analyzed studies demonstrated positive benefits of collaborative peer feedback on the development of professional skills among learners [15]. Manyama et al. demonstrated improved preparation for anatomy dissection sessions and improved confidence with information presentation among students engaged in reciprocal peer teaching [16]. Thus, it can be seen that reciprocal peer feedback offers multiple potential advantages for collaborative learning. This will be instituted via peer review workshops and peer discussions in face-to-face sessions. The peer feedback obtained will be one of the components of the ipsative assessment for each learner. 

The second theory underpinning learning via peer review discussions is the experiential learning theory [17]. By actively engaging learners through peer feedback, peer assessment, and ipsative assessment, learners will be active participants in the learning journey as delineated in Kolb’s learning cycle, which is applicable to various disciplines including the field of medicine [17]. For example, active engagement in the peer review sessions will be a concrete experience and can potentially lead to reflective practice and future experimentation through incorporating peer feedback into future practice. A study by Richard et al. involving orthopaedic residents and residency applicants found that most of them preferred the “converging” learning style, which involves active experimentation, with abstract conceptualization being the second most common learning style [18]. Thus, the planned project initiatives on this teaching programme will feed into this as they will enhance these components of experiential learning. Finally, following the implementation of the project plan, learners will be able to potentially achieve critical evaluation and synthesis skills which are on the higher spectra of Bloom’s taxonomy [19]. 

Possible Challenges and Solutions

Ipsative assessment can be challenging to some learners who are new to this assessment method compared to others who might be familiar with it. As mentioned previously, some “high fliers” might feel it offers no benefit to them and might prefer the traditional high-stakes summative assessments. Theoretically speaking, a learner who has achieved the maximum rating level of 4 that will be employed for each assessed domain may feel there is no higher ceiling to get to. However, the current teaching program is geared towards formative assessment and has no high-stakes summative component. In addition, most learners on the programme are junior doctors for whom it might take some time to achieve level 4 competencies for all domains. Even if a learner achieves level 4 competency for a particular domain, the focus will have to shift towards maintaining this level of competency and applying this in different contexts. These different contexts will have various challenges that can still stimulate learner engagement and minimize the problem of attainment of a learning plateau. Finally, ipsative assessment is already utilized in other areas of the surgical curriculum as mentioned previously and will therefore not be entirely novel when introduced in this programme. 

Some anticipated challenges with peer feedback include perceived novice status, inherent competition among students, and the potential for awarding better marks to friends [20]. The use of grades in peer feedback can potentially accentuate these problems. These anticipated challenges can be overcome by using a model where a structured feedback template is provided and the ratings from peer feedback will be aligned to the ipsative assessment model so that learners can use peer and tutor feedback to assess learning progress [21]. A session will also be devoted at the beginning of the next learning phase towards discussing the structured feedback template, principles of good feedback, and the anticipated pros and cons with the learners. On the other hand, peer assessment like 360-degree multi-source feedback is already an established norm in the field of medical education. This type of feedback is got from colleagues like doctors and nurses who have observed one’s practice and is fed anonymously into an electronic portfolio. The feedback obtained is combined with supervisor and patient feedback during the annual appraisal or review of the competency progression process. Therefore, it is anticipated that peer feedback will not be an entirely novel approach and that learners will accept this as a useful feedback component for learning in the program. 

Another anticipated challenge is a continuation of the implemented changes in this project when a new group of doctors commence rotation within the department. They might not be familiar with the relatively novel changes implemented in the teaching programme as compared to what they are used to. This challenge can be surmounted by ensuring that the programme lead tutor provides an initial session discussing the pedagogical model of the programme and getting feedback from the new group of learners in addition to providing clarifications on any grey areas. In addition, engaging key stakeholders like the consultant educational supervisors and departmental clinical lead can help ensure that the programme initiatives are sustainable. Furthermore, the involvement of the medical education department will aid project sustainability.

Finally, learning is dynamic and complex and no single learning initiative or style will suit every learner. Tutors have varied teaching styles and some doctor-tutors on the programme may lack the advanced teaching pedagogical skills obtainable from a higher education postgraduate certificate or master's degree. There might be some reluctance to accept the ipsative assessment and peer feedback as important components of learning design. These can hopefully be addressed through quarterly tutor engagement forums where discussions will be held on the challenges encountered and reflective feedback gained from the learners. 

## Conclusions

A detailed plan involving two key interventions as part of a quality improvement project to enhance learning on a junior doctor orthopaedic teaching programme has been provided. The planned interventions are the introduction of ipsative assessment and the use of peer review workshops. The reflective practice and skills gained from peers and tutors following the author’s engagement on a master’s level postgraduate certificate in higher education have formed the key reflective feedback pillars for designing this project. Furthermore, a narrative review of the relevant theories and literature evidence has been performed as it relates to this project plan.

The future of medical education will still contain large components of ipsative assessment, which is already widely used for assessment of capability progression on the intercollegiate surgical curriculum. In addition, a problem-based collaborative learning approach is now utilized in many medical schools and peer feedback will become more frequently utilized as a part of this in future. The author looks forward to implementing this project successfully and anticipates that it will be of immense benefit to both tutors and learners and that the principles can be useful for other similar orthopaedic clinical teaching programmes.
